# The complex interplay between chromosome, climatic niche and morphological traits shapes the diversification of *Carex* (Cyperaceae)

**DOI:** 10.1093/aob/mcaf290

**Published:** 2025-11-20

**Authors:** Ana Valdés-Florido, Joan Cuscó-Borràs, Santiago Martín-Bravo, Carmen Benítez-Benítez, José Ignacio Márquez-Corro, Modesto Luceño, Andrew L Hipp, Marcial Escudero

**Affiliations:** Department of Plant Biology and Ecology, Faculty of Biology, University of Seville, Seville 41012, Spain; Department of Molecular Biology and Biochemical Engineering, Universidad Pablo de Olavide, Seville 41013, Spain; Department of Plant Biology and Ecology, Faculty of Biology, University of Seville, Seville 41012, Spain; Department of Molecular Biology and Biochemical Engineering, Universidad Pablo de Olavide, Seville 41013, Spain; Department of Plant Biology and Ecology, Faculty of Biology, University of Seville, Seville 41012, Spain; Department of Molecular Biology and Biochemical Engineering, Universidad Pablo de Olavide, Seville 41013, Spain; Department of Molecular Biology and Biochemical Engineering, Universidad Pablo de Olavide, Seville 41013, Spain; Herbarium and Center for Tree Science, The Morton Arboretum, Lisle, IL 60532, USA; Department of Plant Biology and Ecology, Faculty of Biology, University of Seville, Seville 41012, Spain

**Keywords:** Bioclimatic variables, chromosome evolution, diversification, morphological traits, phylogenetic path analyses

## Abstract

**Background and Aims:**

Shifts in lineage diversification rates, shaped by speciation and extinction, are influenced by morphological, ecological and genetic changes. In this study, we investigate the drivers of diversification in *Carex*, considering chromosome number evolution, bioclimatic variables (temperature and precipitation) and morphological traits (culm and lateral spike unit lengths), along with their evolutionary rates.

**Methods:**

First, we used Bayesian analysis of macroevolutionary mixtures (BAMM) to estimate diversification and trait evolution rates and probabilistic models for chromosome evolution. Phylogenetic path analyses (PPAs) were then used to assess the relative contribution of the predictors to diversification. We tested three different model sets: (1) a set where diversification is predicted by chromosome number, climate and morphology means for each species; (2) a set where diversification is predicted by the evolutionary rates of these three predictors; and (3) a set combining both mean values and evolutionary rates to predict lineage diversification. Additionally, we used the Quantitative State Speciation and Extinction (QuaSSE) model to test the effects of chromosome evolution rates on speciation and extinction.

**Key Results:**

Chromosome number and the rate of chromosome evolution have a weak but significant influence on *Carex* diversification, though it varies across models and clades. Bioclimatic variables and their evolutionary rates also affect diversification, but only indirectly, through their influence on morphology and chromosome evolution.

**Conclusions:**

The subtle but significant influence of chromosome number and its rate of evolution on *Carex* diversification suggests that the remarkable diversity of the genus cannot be explained by a single driver. Instead, it probably results from a complex interplay among bioclimatic, genomic and morphological traits. Notably, the influence of chromosome number is not consistent across all models and clades, highlighting the context-dependent nature of these relationships. Thus, the associations between traits and lineage diversification resist simple linear explanations and may vary across *Carex* lineages.

## INTRODUCTION

Uneven species richness among plant lineages is the result of variation in net diversification rates (i.e. variation in speciation and extinction rates across lineages), which is driven by a complex interplay of intrinsic and extrinsic factors. Among extrinsic factors impacting diversification, ecological conditions have been recognized to play a significant role by creating ecological heterogeneity, driving niche differentiation and facilitating geographical isolation (Wiens and Donoghue, 2004; Hua and Wiens, 2013). Variability in temperature, precipitation and seasonality generates selective pressures that enhance adaptation and speciation. Among intrinsic factors, morphological innovations have also played a crucial role in plant diversification by enabling species to exploit new resources and adapt to varying environmental conditions (Donoghue, 2005). For example, changes such as the evolution of larger leaves can enhance resource acquisition and tolerance to diverse habitats (Wright *et al*., 2004). Additionally, evolutionary transitions between traits (e.g. shifts to more dark-efficient photosynthesis) often open novel ecological opportunities that drive speciation and adaptive radiation across multiple plant lineages (Donoghue, 2005; Edwards, 2019).

Chromosome evolution – particularly polyploidy (i.e. whole genome duplication, WGD) and dysploidy (i.e. variations in chromosome number without variation of ploidy level) – also shape diversification rates (Escudero *et al*., 2012; Mandáková and Lysak, 2018; Márquez-Corro *et al*., 2021; Zhan *et al*., 2021; Tribble *et al*., 2025). Polyploidy can lead to increased genetic diversity and also the development of novel traits, both of which can facilitate plant speciation (e.g. Soltis *et al*., 2009; Heslop-Harrison *et al*., 2023). Dysploidy is more generally expected to influence reproductive isolation, potentially (but not always) leading to speciation (Freyman and Höhna, 2018). As a consequence, high rates of diversification have been associated with holocentricity (i.e. having chromosomes that lack localized centromeres; Márquez-Corro *et al*., 2018). In these chromosomes, centromeric regions with kinetochoric activity are distributed along the whole chromosome, making them prone to dysploidy by way of fissions and fusions (Guerra, 2016; Lucek *et al*., 2022).

While studies have often examined the role of chromosome number evolution, morphological trait changes and environmental factors independently, integrative approaches that assess their combined effect on diversification remain relatively scarce. Rather than acting in isolation, chromosomal and genome evolution may influence changes in morphological traits and, in turn, be shaped by shifts in ecological conditions (e.g. Elliott *et al*., 2022; Moraes *et al*., 2022), with important implications for diversification. For instance, chromosome numbers have been significantly associated with temperature variables (Las Peñas *et al*., 2024), as well as with morphology and climatic niche (Márquez-Corro *et al*., 2021). Although environmental and morphological traits often explain only a small proportion of chromosomal variation, evidence suggests that their interaction can shape long-term macroevolutionary patterns across clades (Román-Palacios *et al*., 2020).

The genus *Carex* (Cyperaceae) is one of the most diversified genera among angiosperms, with more than 2000 accepted species (Roalson *et al*., 2021; Jiménez-Mejías *et al*., 2025). *Carex* is a nearly cosmopolitan genus, with its greatest diversity in the boreo-temperate Northern Hemisphere and to a lesser extent in the temperate regions of the Southern Hemisphere (Martín-Bravo *et al*., 2019). The extensive distribution of *Carex* has prompted numerous biogeographical and ecological studies at both macro- and microevolutionary scales (e.g. Waterway *et al*., 2009; Pender, 2016; Spalink *et al*., 2016*a*, *b*; Benítez-Benítez *et al*., 2018; Valdés-Florido *et al*., 2025). Although *Carex* maintains morphological coherence as a genus, morphological variation may influence competitive interactions as well as reproductive strategies. In fact, Márquez-Corro *et al*. (2021) found recurrent shifts of both culm height and lateral inflorescence length independently across the phylogeny of *Carex*, raising questions as to whether these trait transitions might have triggered speciation by facilitating adaptation to new habitats. Moreover, previous studies point towards bioclimatic niche evolution shaping diversification at shallow evolutionary scales (Villaverde *et al*., 2017; Benítez-Benítez *et al*., 2018, 2021*a*). However, *Carex* seems to have generally retained a preference for cold-temperate habitats (Benítez-Benítez *et al*., 2021*b*), suggesting that large-scale bioclimatic niche shifts might not represent the primary driver of macroevolutionary diversification in *Carex*.

By contrast, *Carex* exhibits high variability in chromosome number, ranging from 2*n* = 10 to 2*n* = 132 (Tanaka, 1939; Davies, 1956; Roalson, 2008; Hipp *et al*., 2009), due mostly to dysploidy rather than polyploidy (Márquez-Corro *et al*., 2018). The high rates of chromosome number evolution and the presence of holocentric chromosomes in *Carex* have made it a focal point for studying diversification patterns (e.g. Escudero *et al*., 2013; Márquez-Corro *et al*., 2021; Tribble *et al*., 2025). In a recent study, Tribble *et al*. (2025) developed the ChromoHiSSE model to determine whether chromosome number changes drive *Carex* cladogenesis or if other hidden factors contribute to its diversification. Their analyses recovered two different modes of chromosomal evolution within the phylogeny. In certain clades, dysploidy occurs much more frequently, particularly through anagenetic changes, and chromosome number shifts appear to be the primary drivers of cladogenetic events. The ‘last-straw’ hypothesis has been proposed to explain how the accumulation of chromosomal changes through dysploidy can lead to reproductive isolation and ultimately promote cladogenesis (Baker and Bickham, 1986; Whitkus, 1988; Escudero *et al*., 2016; Tribble *et al*., 2025). In contrast, some clades exhibit much lower frequencies of dysploidy, which is not associated with cladogenesis, suggesting that unobserved factors may play a hidden role in diversification (Tribble *et al*., 2025). Their work thus shows that chromosome evolution is key to explaining sedge diversification, along with some other unmeasured ‘hidden states’ (the Hi in ChromoHiSSE).

In this study, we aim to identify the key predictors of species diversification. Specifically, we explore the role of chromosome evolution, bioclimatic variables, and vegetative and reproductive morphological trait variation, along with their respective rates of evolution, in shaping diversification rates. Our general hypothesis is that the diversification in *Carex* is driven by the interplay among evolution of chromosome number, bioclimatic niche and morphological traits. More specifically, we expect that: (1) chromosome number and the rate of chromosomal change will be correlated with both morphological evolution and diversification rates; (2) the rate and extent of bioclimatic niche evolution will help to explain patterns of morphological and chromosomal evolution as well as diversification rates; and (3) the rate and magnitude of evolutionary change in key vegetative and reproductive morphological traits will be associated with diversification rates.

## MATERIAL AND METHODS

### Datasets

We used the phylogeny of Márquez-Corro *et al*. (2021), originally based on that from Martín-Bravo *et al*. (2019), which encompasses 66 % of the diversity of the genus *Carex*. Chromosome number and morphological and bioclimatic trait datasets were also retrieved from Márquez-Corro *et al*. (2021) . From the 19 climate variables available in the WorldClim database (https://www.worldclim.org/), we selected BIO1 (Annual Mean Temperature), BIO4 (Temperature Seasonality, standard deviation ×100), BIO7 (Temperature Annual Range) and BIO12 (Annual Precipitation) to best represent climatic variation, following Márquez-Corro *et al*. (2021). For morphological traits, culm length and lateral spike unit length were chosen as representative measures of vegetative and reproductive variation, also based on Márquez-Corro *et al*. (2021).

### Diversification rates and the evolutionary rates of morphological and bioclimatic traits

Bayesian analyses of macroevolutionary mixtures (BAMM v.2.5; Rabosky *et al*., 2013, 2014; Shi and Rabosky, 2015) were used to model the evolution of selected bioclimatic data (BIO1, BIO4, BIO7, BIO12) and traits (culm length and lateral spike unit length) across the *Carex* phylogeny. BAMM implements reversible-jump Markov chain Monte Carlo (MCMC) to automatically explore the full range of possible models of lineage diversification and trait evolution, limited by the inclusion or exclusion of all considered model parameters. Our diversification analyses allowed for rate shifts in speciation and extinction, as well as changes in diversification rates over time within each subtree model (modelling gradual evolutionary dynamics in speciation rates in each subtree). Similarly, trait evolution analyses allowed shifts in the rates of continuous trait evolution and changes within each regime. We ran BAMM for 10 million generations per analysis, conducting eight analyses in total. We used the R package *coda* (Plummer *et al*., 2006) to assess MCMC convergence and *BAMMtools* (Rabosky *et al*., 2014) to process the output and summarize model parameters with the highest posterior probabilities. The rates of bioclimatic and morphological evolution for each species (or subspecies) were extracted from the evolutionary rates estimated at the terminal branches of the phylogeny (i.e. external branches or tips).

Instead of estimating speciation and extinction rates from the pruned phylogeny of Márquez-Corro *et al*. (2021), which has a sampling of around 40 % of the species, we directly retrieved these data from Martín-Bravo *et al*. (2019), who estimated diversification rates using BAMM, based on the original phylogeny including approximately 70 % of extant *Carex* species (this is the same phylogeny we have used in this study but before pruning the tips without chromosome number or trait data). Net diversification rates (speciation minus extinction rates) for each species (or subspecies) were also extracted from rates modelled at the terminal branches of the phylogeny.

Finally, rates of chromosome evolution were estimated using ChromEvol (Glick and Mayrose, 2014). We implemented a model in which chromosome number could change through single gains and losses (dysploidy), with rates allowed to vary linearly with chromosome number. Polyploidy, although very infrequent in *Carex* (Hipp *et al*., 2009), was also allowed. For this analysis, the subgenus *Siderostictae* was included, but then excluded from downstream analyses (changes in this clade, sister to the rest of the genus, are restricted to polyploid changes without dysploidy). In addition, we used a new implementation of the ChromEvol model that allows for shifts in the rate of chromosome evolution (Shafir *et al*., 2023, 2025). The minimum number of tips required to consider a new model with different rates of chromosome evolution was set to 10, and the maximum number of distinct models allowed was set to 40. Rates of dysploidy for each taxon were calculated based on both constant and linear (chromosome number-dependent) rates of gains or losses, as determined by the fitted model allowing for rate heterogeneity.

### Phylogenetic path analyses

We used phylogenetic path analyses (PPAs; Hardenberg and González-Voyer, 2013; van der Bijl, 2018) to understand which are the most important predictors of species diversification, measured as diversification rates at the tips of the phylogeny from BAMM analyses, and explore the relationships among the considered predictors. We tested two categories of predictors: (1) *species means* of chromosome number, bioclimatic variables and morphological traits; and (2) *evolutionary rates*, including rates of chromosome number evolution, bioclimatic trait evolution and morphological trait evolution (all estimated at the tips of the phylogeny inferred by BAMM analyses). We performed these analyses using the R package *phylopath* (van der Bijl, 2018), which implements PPA as described by Hardenberg and González-Voyer (2013). The script and datasets used are available in Valdés-Florido *et al*. (2025). This approach allows us to evaluate and compare causal models using observational data while accounting for phylogenetic relationships by fitting multiple phylogenetic regressions corresponding to each path in the model. We tested multiple alternative models in which diversification is influenced by different combinations of predictor variables, which are described below.

#### Analyses considering only rates of evolution (48 models)

**Fig. 1.**
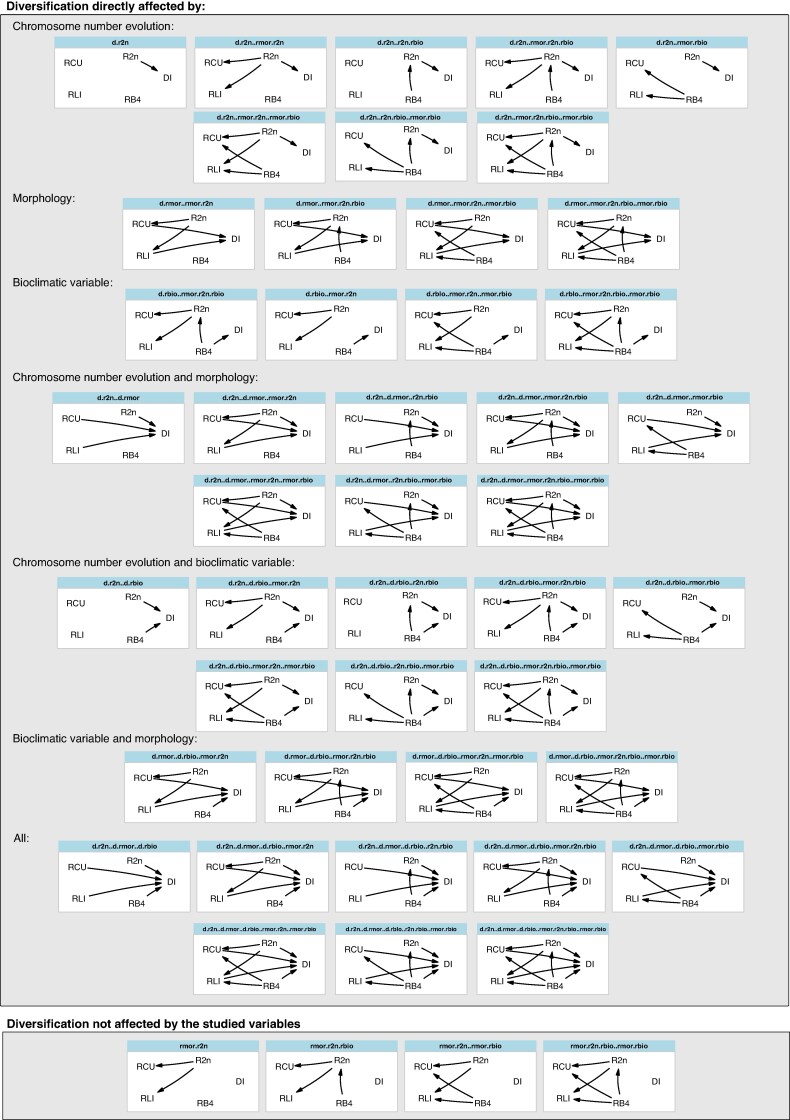
Set of 48 tested models representing hypotheses of different rates influencing diversification in *Carex*. *RB4* corresponds to the evolutionary rate of BIO4 (temperature seasonality), *R2n* corresponds to the evolutionary rate of chromosome number evolution, *RLI* indicates the evolutionary rate of lateral spike unit length, *RCU* indicates the evolutionary rate of culm length and *DI* indicates diversification rates. In the model code, the predictor influencing diversification is included after *d*.

We initially performed PPA considering only evolutionary rates for chromosome number, morphological traits and bioclimatic variables as predictors of diversification rates. First, we defined six simple models centred on the role of chromosome rates of evolution: (1) diversification rates are predicted by chromosome rates of evolution; (2) morphological rates of evolution are predicted by chromosome rates of evolution; (3) both diversification rates and rates of morphological evolution are predicted by rates of chromosome number evolution; (4) diversification rates are predicted by rates of chromosome evolution, which are predicted by rates of climate evolution; (5) rates of morphological evolution are predicted by rates of chromosome evolution that are predicted by rates of climate evolution; and (6) both diversification rates and rates of morphological evolution are predicted by rates of chromosome number evolution, which are predicted by rates of climate evolution. These six models were expanded to include 18 additional models in which diversification rates are directly predicted by rates of morphological evolution (six additional models), rates of bioclimatic evolution (six additional models), or rates of morphological and bioclimatic evolution (six additional models). Finally, these 24 models were doubled by considering that morphological rates of evolution could be predicted by bioclimatic rates of evolution. In total, 48 different models were compared (see Fig. 1).

#### Analyses considering only species means but not rates of evolution (48 models)

We also analysed the same 48 models as before, but with species means of chromosome number, morphological traits and bioclimatic variables instead of their evolutionary rates (Supplementary Data Fig. S1).

#### Analyses considering rates only, species means only, and both (332 models)

We used the previous 48 models considering only rates of evolution and the 48 models based on species means, as described previously. Similarly, we expanded the models based on species means by including rates of chromosome number evolution. This resulted in 47 models, as a model in which diversification rates are proportional to chromosome number and chromosome number rates had already been included in the previous one. We also used 47 additional models similar to those considering only species means, but with all models focusing on chromosome number replaced by models considering only rates of chromosome evolution (without chromosome number). Furthermore, we considered 47 more models in which chromosome number rates were replaced by chromosome number, excluding rates of evolution. Finally, we tested 47 additional models that included all species means (morphological traits, bioclimatic variables and chromosome number) alongside their respective evolutionary rates as predictors.

The *phylopath* package was used to evaluate the models. Models were ranked using the C-statistic information criterion corrected for small sample sizes (CICc) and the difference in CICc with the top model (delta_CICc). For model selection, we performed full model averaging on all models within 2 units of the minimum CICc. Path averaging was conducted for all paths in all models, irrespective of whether a model includes a given path, to avoid coefficient biases away from zero assuming that the coefficients (and their variance) for absent paths to be zero. The coefficient calculated by *phylopath* is a standardized coefficient that ranges from −1 (the highest inverse effect) to 1 (the largest direct effect), with 0 indicating no effect.

### Quantitative State Speciation and Extinction analysis (QuaSSE)

We used the Quantitative State Speciation Extinction model (QuaSSE) as implemented in the R package *diversitree* v.0.9-14 (FitzJohn, 2010) to assess the effect of chromosome number rates of evolution as inferred from the ChromEvol model on speciation and extinction rates. Following the approach of Márquez-Corro *et al*. (2021), who applied QuaSSE to chromosome number rather than its rate of evolution, we evaluated all possible model combinations describing constant, linear, sigmoid or unimodal relationships between chromosome number evolution rates and speciation or extinction events.

## RESULTS

### Rates of morphological and bioclimatic evolution

All the analyses reached stationarity and convergence. The analyses of bioclimatic variables displayed a very stable evolution rate with no changes for three of the four analysed variables (BIO1, BIO7 and BIO12) and only two shifts in BIO4 (Supplementary Data Fig. S2). However, the rate of morphological evolution displayed many shifts (26 shifts in the rate of culm evolution – Fig. S3 – and 95 shifts in the evolution of lateral spike unit length –Fig. S4).

#### Rates of chromosome evolution

**Fig. 2.**
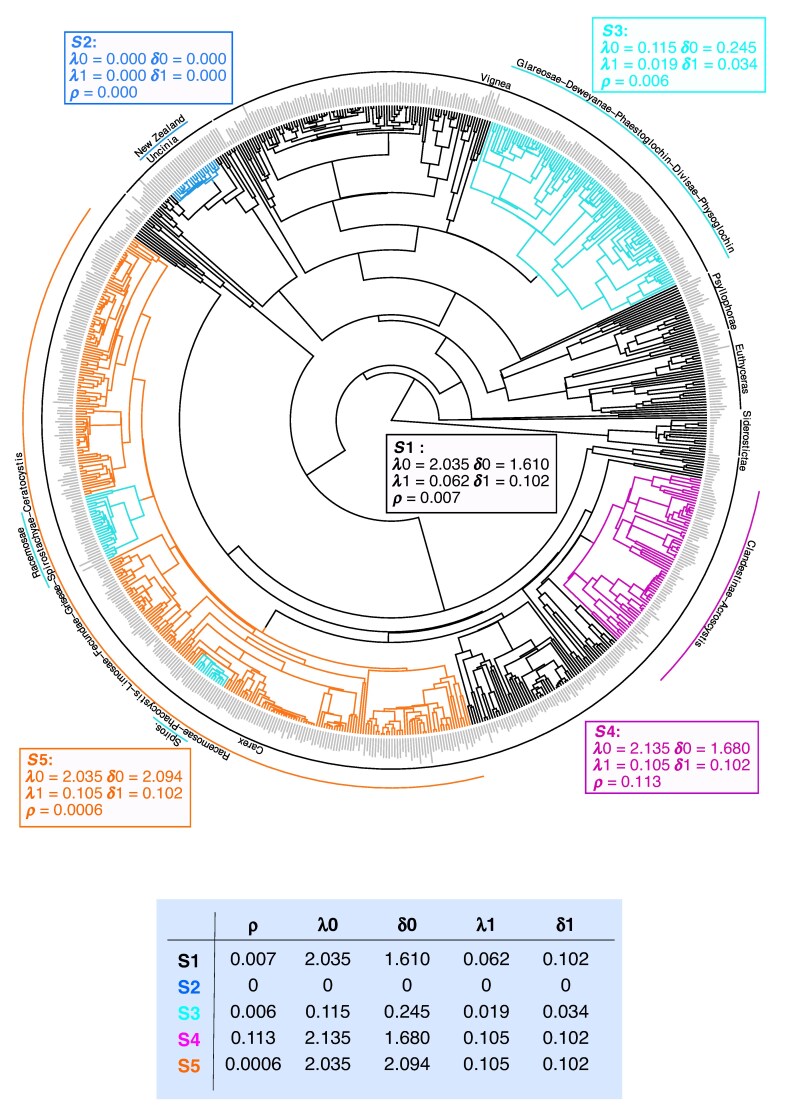
Phylogeny of the genus *Carex* representing the models of chromosome evolution (models S1–S5) and a summary table summarizing the estimated rates, inferred using ChromEvol. ρ corresponds to duplication rates, λ0 and δ0 correspond to ascending and descending dysploidy, respectively, and λ1 and δ1 correspond to ascending and descending dysploidy rates in relation to haploid chromosome numbers, respectively. Rates are shown in events my^−1^  *n*^−1^.

The analysis of chromosome number evolution showed six shifts in the model of chromosome evolution, resulting in five different models with varying rates of ascending and descending dysploidy, as well as polyploidy (Fig. 2). These models are numbered from 1 to 5 in relation to their significance and here we detail them in that order.

Model 1 (or background model) shows a very low polyploidy rate [ρ = 0.007 events per million years (my^−1^)] and very high ascending (λ0 = 2.035 events my^−1^) and descending (δ0 = 1.610 events my^−1^) dysploidy rates that vary in relation to haploid chromosome number (λ1 = 0.062 events my^−1^  *n*^−1^ and δ1 = 0.102 events my^−1^  *n*^−1^).

Model 2 was inferred for the New Zealand Uncinia clade where all rates are equal to zero.

Model 4 is inferred for a clade that contains the sections *Clandestinae* and *Acrocystis*, and other related clades. Here, a significant increase of polyploidy is inferred (ρ = 0.113 events my^−1^) and rates of dysploidy are higher than in model 1 (ascending: λ0 = 2.135 events my^−1^ + λ1 = 0.105 events my^−1^  *n*^−1^, and descending: δ0 = 1.680 events my^−1^ + δ1 = 0.102 events my^−1^  *n*^−1^).

Model 5 is inferred for a large clade in the subgenus *Carex*, which includes large sections such as *Racemosae*, *Phacocystis*, *Limosae*, *Fecundae*, *Griseae*, *Spirostachyae* and *Ceratocystis*, among other sections and related clades. In this model, a significant decrease of duplication rates (ρ = 0.0006 events my^−1^) and a significant increase of dysploidy rates (ascending: λ0 = 2.035 events my^−1^ + λ1 = 0.105 events my^−1^  *n*^−1^, and descending: δ0 = 2.094 events my^−1^ + δ1 = 0.102 events my^−1^  *n*^−1^) compared to model 1 are inferred.

Model 3 was inferred in convergence in three different clades of the phylogeny. First, it is inferred in a large clade within the subgenus *Vignea*, including sections *Glareosae*, *Deweyanae*, *Phaestoglochin*, *Divisae* and *Physoglochin*, and other related clades. Second, it is inferred in section *Racemosae* within the subgenus *Carex*. Third, it is inferred in a subclade of section *Spirostachyae* that includes *Carex* gr. *laevigata* and the clade from Eastern Tropical Africa. The first inference represents a shift from model 1, while the second and third correspond to shifts from model 5. Model 3 infers a rate of duplication (ρ = 0.006 events my^−1^) similar to that of model 1, yet significantly higher than that estimated in model 5. Finally, a highly significant deceleration in both ascending (λ0 = 0.115 events my^−1^ + λ1 = 0.019 events my^−1^  *n*^−1^) and descending (δ0 = 0.245 events my^−1^ + δ1 = 0.034 events my^−1^  *n*^−1^) dysploidy rates is inferred, in comparison with both models 1 and 5.

### Phylogenetic path analyses

**Fig. 3.**
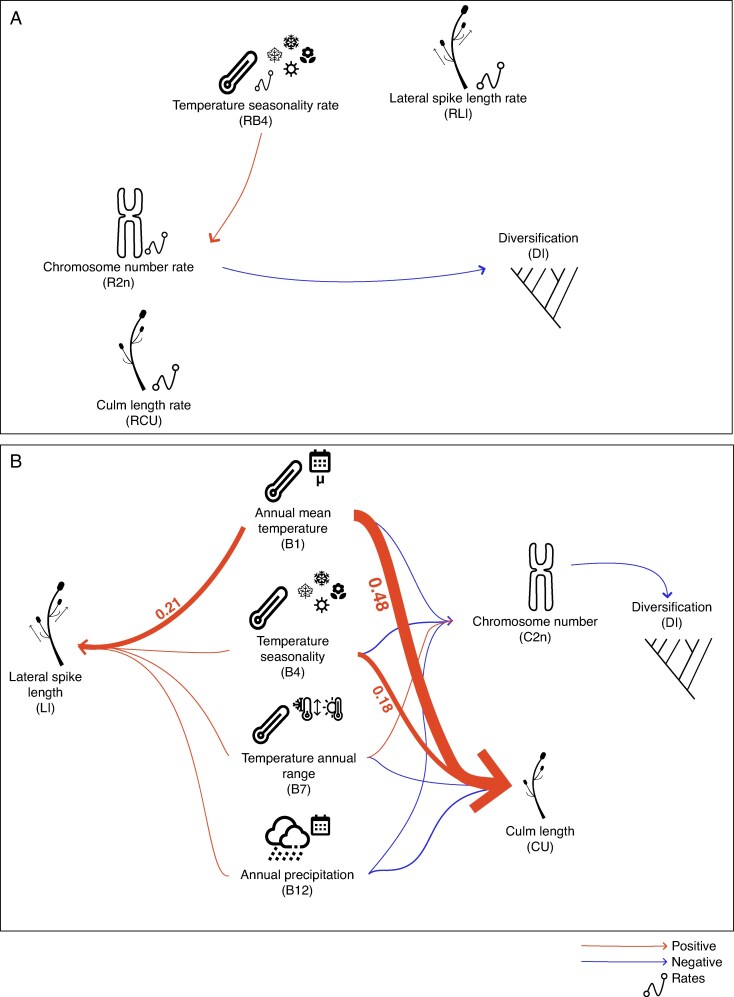
(A) Representation of the full average best model including only evolutionary rates, as inferred from the phylogenetic path analyses. *RB4* represents the evolutionary rate of BIO4 (temperature seasonality), *R2n* corresponds to the evolutionary rate of chromosome number, *RLI* indicates the evolutionary rate of lateral spike unit length, *RCU* refers to the evolutionary rate of culm length and *DI* represents diversification rates. Arrow thickness is proportional to the values. Coefficients <0.1 are not shown but can be found in Supplementary Data Table S1. (B) Full average best model only with variables, as inferred from the phylogenetic path analyses. *B1*, *B4*, *B7* and *B12* correspond to the studied bioclimatic variables (BIO1 – annual mean temperature, BIO4 – temperature seasonality, BIO7 – temperature annual range, BIO12 – annual precipitation, respectively). *LI* corresponds to lateral spike unit length, *CU* to culm length, *C2n* to chromosome number evolution and *DI* to diversification rates. Arrow thickness is proportional to the values. Coefficients <0.1 are not shown but can be found in Supplementary Data Table S2.

When only rates were considered (48 models that contain 250 phylogenetic regressions, of which 30 are unique, Supplementary Data Fig. S5A), the best-fitting model was the null model (i.e. the no-predictor model). The second-best model, with only a difference of 0.003 units of CICc, was d.r2n..r2n.rbio, in which chromosome evolution rates affect diversification while temperature seasonality rates have an effect on chromosome evolution rates. Model averaging (Fig. 3A; Table S1) indicated that rates of climate evolution have a slight influence on diversification rates and chromosome evolution rates, but standardized regression coefficients are smaller than |0.01|. This model also suggests a weak effect of chromosome evolution rates on diversification rates, while morphological rates neither influence nor are influenced by other rates.

When only variables were considered (48 models that contain 844 phylogenetic regressions, of which 66 are unique; Supplementary Data Fig. S5B), the best-fitting model was d.2n..2n.bio..mor.bio. This model implies a direct effect of bioclimatic variables on morphology and on chromosome number evolution, as well as a direct effect of chromosome number evolution on diversification (Fig. S1). Additionally, within 2 units of CICc, another model is inferred (i.e. d.2n..mor.bio), which differs only by not considering the effect of bioclimatic variables on the number of chromosomes. Model averaging (Fig. 3B; Table S2) indicates a weak negative effect of chromosome evolution on diversification. Moreover, it reveals both positive and negative effects of bioclimatic variables on morphological traits and chromosome evolution. The strongest effects of this model are those of annual mean temperature (BIO1), which are positive for both culm length (CU) and lateral inflorescence unit length (LI; see Table S2 for more details).

**Fig. 4.**
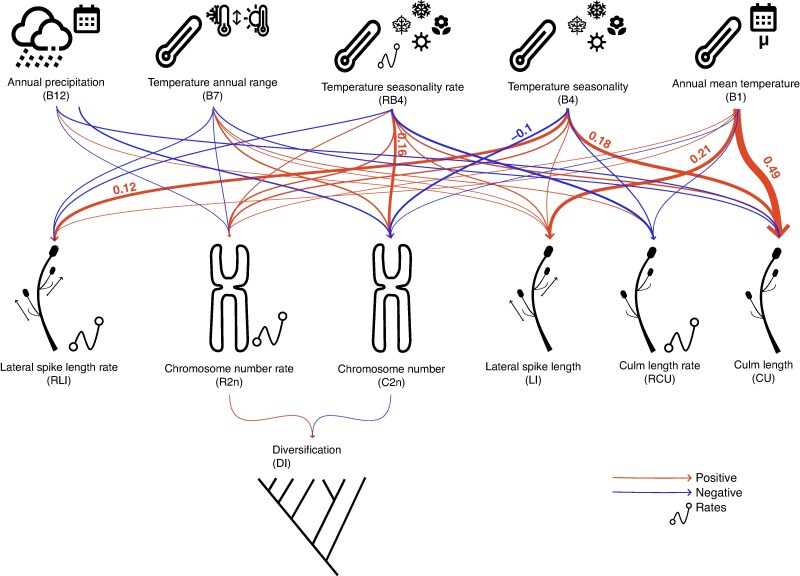
Visualization of the full average best model, considering both variables and their evolutionary rates, as inferred from the phylogenetic path analyses. In the upper part, bioclimatic variables are represented: *B1* (annual mean temperature), *B4* (temperature seasonality), *B7* (temperature annual range) and *B12* (annual precipitation). *RB4* indicates the evolutionary rates of B4. *LI* and *RLI* correspond to lateral spike unit length and its evolutionary rate, respectively. *CU* and *RCU* indicate culm length and its evolutionary rate, respectively. Finally, *C2n* and *R2n* represent chromosome evolution and its rate, respectively; *DI* indicates diversification rates. Arrow thickness is proportional to the values. Coefficients <0.1 are not shown but can be found in Supplementary Data Table S3.

When both rates and variables were considered (332 models that contain 18 112 phylogenetic regressions, of which 655 are unique; Supplementary Data Fig. S5C), the best-fitting model was d.b2n..b2n.bbio..bmor.bio, in which diversification rates are predicted by both chromosome number and its evolutionary rates. The evolution of bioclimatic variables also influences morphological evolution (including both traits and their rates) and chromosome evolution (both number and rates). The full averaged best model (Fig. 4) indicated that diversification rates are affected by the number of chromosomes and its evolutionary rates. Furthermore, bioclimatic evolution has an impact on both morphological evolution and chromosome number evolution. Culm length is mostly affected by BIO1 – annual mean temperature (0.49, CI = 0.38–0.60), BIO4 – temperature seasonality (0.18, CI = −0.14 to 0.50), and to a lesser extent by RBIO4, BIO7 –temperature annual range – and BIO12 – annual precipitation (see Table S3 for more details). Lateral inflorescence unit length is also predicted by bioclimatic variables (see Table S3 for more details).

### Quantitative State Speciation and Extinction analysis (QuaSSE)

The best model (OU.hump.sigmoid; see Supplementary Data Table S4) was a very complex model in which chromosome evolution rates follow an Ornstein–Uhlenbeck (OU) model with a hump-shaped relationship with speciation rates and a sigmoid relationship with extinction rates (Fig. S6). This model was significantly better than the second-best model (OU.hump.linear). This second model had an Akaike information criterion (AIC) approximately 34 units higher. Under this best-fit model, speciation rates remain relatively constant (<2 lineages my^−1^), exhibiting a slight decrease at intermedium rates of chromosome evolution before returning to their initial values. Extinction rates, which are slightly lower than speciation rates, also show a slight decrease at intermediate chromosome evolution rates. As a result, net diversification rates (<1 lineage my^−1^) display a slight increase at medium rates of chromosome evolution, coinciding with lower rates of both speciation and extinction (Fig. S6).

## DISCUSSION

In this work we tested the effect of bioclimatic variables, vegetative and reproductive morphological traits and chromosome number, including their rates of evolution, as key predictors of the diversification of *Carex*. Previous phylogenetic studies (e.g. Waterway *et al*., 2009; Escudero *et al*., 2010, 2012; Pender, 2016; Spalink *et al*., 2016*a*, *b*; Benítez-Benítez *et al*., 2018, 2021*b*; Martín-Bravo *et al*., 2019; Tribble *et al*., 2025; Valdés-Florido *et al*., 2025) point to the potential importance of ecological, biogeographical and chromosomal processes in sedge lineage diversification. Our analyses tease apart the direct and indirect effects of morphological traits, climate and chromosome number – all implicated in earlier studies as potential drivers of *Carex* diversification – on the evolutionary success of the genus. By integrating these factors within a phylogenetic framework using phylogenetic path analyses, we demonstrate the relative contributions of these potential diversification drivers to the diversification of this megadiverse genus.

Our results suggest that chromosome evolution is a heterogeneous process in *Carex*. Using probabilistic models that allow for shifts in the model of chromosome evolution, we inferred a complex model with six shifts in the mode of chromosome evolution and five different evolutionary models identified (one of the models originated independently three times). This result, although complex, implies a significantly smaller number of shifts than when we model chromosome evolution in *Carex* as a multiple OU model (Márquez-Corro *et al*., 2021). In general, we have inferred significantly very low rates of polyploidy, consistent with previous findings for the genus *Carex* (Hipp *et al*., 2009), except for model 4 inferred for a clade within the subgenus *Carex* that includes sections *Clandestinae*, *Acrocystis* and related lineages with rates of polyploidy similar to or even higher than the rest of the angiosperms (Zenil-Ferguson *et al*., 2017; Carta *et al*., 2020). The prevalence of polyploidy in section *Acrocystis* had already been suggested by Więcław *et al*. (2020). As previously known, the rates of dysploidy in true sedges (genus *Carex*), which, like the rest of Cyperaceae, have holocentric chromosomes, are incredibly high (Shafir *et al*., 2023). When analysing the family Cyperaceae as a whole using this model that allows for heterogeneity in rates of chromosome evolution, changes in the rates of dysploidy were already inferred along the phylogeny (Shafir *et al*., 2023). Nevertheless, our findings suggest not only consistently high dysploidy rates but also substantial rate variation among clades without dysploidy (see model 2) or clades with a rate an order of magnitude lower than the rest of the true sedges (see model 3). Here, the current chromosome number appears to be a very important factor to determine rates of dysploidy.

PPA further suggests that chromosome evolution plays an important role in shaping diversification patterns in *Carex* (Figs 3 and 4; Supplementary Data Fig. S5), although the inferred effect is weak. When focusing only on evolutionary rates, we found that diversification is associated with rates of chromosome evolution, not with the rates of change in morphological or bioclimatic traits (Fig. 3A; Fig. S5A). When analysing only species means, bioclimatic variables appear to influence both morphology and chromosome evolution, while lineage diversification is linked exclusively to chromosome number evolution (Fig. 3B; Fig S5B). Finally, in the most complex model, which includes both species means and their evolutionary rates, we found that both morphological traits and chromosome evolution are affected by bioclimatic variables (both evolutionary rates and species means), but the diversification of the genus remains directly influenced only by mean chromosome number and rates of chromosome evolution (Fig. 4; Fig. S5C). Thus, the three model sets indicate that chromosome evolution may contribute directly to diversification in *Carex*, albeit weakly. Moreover, some models suggest that morphological traits and bioclimatic variables may affect diversification indirectly, through their influence on chromosome evolution (Fig. 4).

Previous studies have also highlighted chromosome evolution as an important driver of diversification in *Carex* (e.g. Escudero *et al*., 2010; Márquez-Corro *et al*., 2018, 2024; Tribble *et al*., 2025). Unlike monocentric chromosomes, where kinetochoric activity is restricted to a localized centromere, holocentric chromosomes, such as those of *Carex*, have kinetochoric activity in centromeric regions along the entire chromosomes (Marques *et al*., 2015; Márquez-Corro *et al*., 2018). This structure allows fragments to segregate normally during meiosis, facilitating chromosome number variation via dysploidy (Faulkner, 1972) and potentially driving speciation (Márquez-Corro *et al*., 2018). Although chromosome variation through dysploidy occurs more frequently in holocentric clades than in monocentric ones, diversification rates appear not to differ systematically between monocentric and holocentric clades (Márquez-Corro *et al*., 2018). Shifts in diversification rates might nonetheless result from the interplay between clade-specific evolutionary selection pressures and the advantages/disadvantages conferred by either monocentric or holocentric chromosome structures (Márquez-Corro *et al*., 2018).

A potential interaction between chromosome structure and some additional (unidentified) factors was reported in previous work. When jointly modelling chromosome evolution and cladogenesis in true sedges, Tribble *et al*. (2025) found that models including a hidden state fit the data best, such that in some clades, rates of chromosome evolution were low and unrelated to cladogenesis, while in others, chromosome evolution occurred at high rates, especially anagenetically, and most cladogenetic events were associated with chromosome number changes. These heterogeneous relationships between chromosome number and cladogenesis may explain the weak effect of chromosome number on diversification rates inferred in our PPAs. Moreover, the relationship between rates of chromosomal evolution and diversification rates using QuaSSE follows a sigmoid distribution, with diversification rates positively correlated with rates of chromosome evolution (Supplementary Data Fig. S6). This non-linear relationship supports Tribble *et al.*’s ‘last-straw’ hypothesis for chromosomal speciation in true sedges, which suggests that chromosomal changes may accumulate gradually until a final rearrangement that triggers speciation. Speciation in *Carex* indeed seems to be driven by the cumulative effect of chromosomal changes.

Although chromosome evolution appears to have the dominant direct effect on lineage diversification in *Carex*, morphological traits and bioclimatic variables also have an indirect effect (Fig. 4). In our most complex model, morphological traits (i.e. culm length and lateral inflorescence unit length) appear to be strongly influenced by bioclimatic variables (Fig. 4; Supplementary Data Fig. S5C), which have been shown in previous work to influence plant morphology (Aslam *et al*., 2022), including in *Carex* (e.g. Stenström *et al*., 2002; Wang *et al*., 2023). For example, in species such as *C. bigelowii*, *C. ensifolia* subsp. *arctisibirica*, *C. lugens* or *C*. *stans*, temperature fluctuations affect traits like shoot height, stomatal density and leaf width (Stenström *et al*., 2002). Such indirect impact of bioclimatic variables on diversification aligns with the broad geographical and ecological distribution of *Carex*, which extends across diverse habitats worldwide (Martín-Bravo *et al*., 2019). This environmental heterogeneity probably drives morphological adaptations, leading to ecological differentiation and niche specialization (Villaverde *et al*., 2017; Benítez-Benítez *et al*., 2018). These processes can promote reproductive isolation and ultimately drive speciation and diversification. Therefore, while chromosome evolution may contribute substantially to diversification in *Carex* (Fig. 4), its relative importance appears to be context-dependent (Fig. 2) and potentially mediated by ecological conditions and morphological evolution, which also play an important indirect role in shaping the macroevolutionary patterns of the genus.

### Final remarks

Even with relatively complex PPA models, the effects of chromosome number evolution and its rate in *Carex* diversification are challenging to detect and subtle, suggesting a complex interplay among bioclimatic and morphological traits, chromosome evolution, and lineage diversification rates. These relationships are, moreover, non-linear and variable across the *Carex* phylogeny. Our study demonstrates nonetheless that, while morphological traits and environmental variables play an indirect role in lineage diversification, chromosome evolution is a direct driver of *Carex* speciation and extinction. These findings highlight the importance of considering both genome rearrangements and ecological context when exploring the macroevolutionary processes shaping biodiversity. Future research should also explore the temporal context of diversification, as events such as the Quaternary glaciations may have played a significant role in shaping diversification patterns within the genus.

## Supplementary Material

mcaf290_Supplementary_Data

## Data Availability

Scripts and datasets are available at Zenodo: doi:10.5281/zenodo.16573067

## SUPPLEMENTARY DATA

Supplementary data are available at *Annals of Botany* online. Figure S1. Set of 48 tested models representing hypotheses of different variables influencing diversification in *Carex*. *B1*, *B4*, *B7* and *B12* correspond to the studied bioclimatic variables (BIO1 – annual mean temperature, BIO4 – temperature seasonality, BIO7 – temperature annual range, and BIO12 –annual precipitation, respectively). *LI* corresponds to lateral spike unit length, *CU* to culm length, *C2n* to chromosome number evolution and *DI* to diversification rates. In the model code, the predictor influencing diversification is included after *d*. Figure S2. Phylogeny showing shifts in evolutionary rates of bioclimatic variables from BAMM analysis: (A) BIO1 (annual mean temperature), (B) BIO4 (temperature seasonality), (C) BIO7 (temperature annual range) and (D) BIO12 (annual precipitation). Figure S3. Phylogeny showing shifts in the rate of culm evolution from BAMM analysis. Figure S4. Phylogeny showing shifts in the evolution of lateral unit length from BAMM analysis. Figure S5. (A) Models support using only evolutionary rates, assessed through phylogenetic path analyses. (B) Model support using only variables, assessed through phylogenetic path analyses. (C) Model support using both variables and evolutionary rates, assessed through phylogenetic path analyses. Bars represent model weights, with colour indicating whether models fall within 2 CICc units. Figure S6. Relationship between chromosome number evolution and speciation (λ), extinction (μ) and net diversification rates, inferred by the QuaSSE method. Table S1. Estimated coefficient for the full average best model among the 48 models with only rates of evolution, inferred through phylogenetic path analyses. *DI* corresponds to diversification rates, *R2n* represents the rate of chromosome evolution, *RCU* represents the evolutionary rate of the culm length, *RLI* corresponds to the evolutionary rate of lateral spike unit length and *RB4* represents the evolutionary rate of BIO4 (temperature seasonality). The highest coefficient is in bold type. Table S2. Estimated coefficient for the full average best model among the 48 models with only variables of evolution, inferred through phylogenetic path analyses. *B1*, *B4*, *B7* and *B12* correspond to the studied bioclimatic variables (BIO1 – annual mean temperature, BIO4 – temperature seasonality, BIO7 –temperature annual range, BIO12 – annual precipitation, respectively). *LI* corresponds to lateral spike unit length, *CU* to culm length, *C2n* to chromosome number evolution and *DI* to diversification rates. The highest coefficient is in bold type. Table S3. Estimated coefficient for the full average best model among the 332 models with both variables of evolution and their rates of evolution, inferred through phylogenetic path analyses. *B1*, *B4*, *B7* and *B12* correspond to the studied bioclimatic variables (BIO1 – annual mean temperature, BIO4 – temperature seasonality, BIO7 – temperature annual range, BIO12 – annual precipitation-), and *RB4* indicates the evolutionary rate of BIO4 – temperature seasonality. *LI* and *RLI* correspond to lateral spike unit length and its evolution rate, respectively. *CU* and *RCU* indicate culm length and its evolution rate, respectively. Finally, *C2n* and *R2n* represent chromosome evolution and its rate, respectively; *DI* indicates diversification rates. The highest coefficient is in bold type. Table S4. Model comparison results from QuaSSE analyses evaluating the relationship between trait evolution and diversification. The best-supported model based on AIC scores is in bold type.
