# Supplementary material for: The complex interplay between chromosome, climatic niche and morphological traits shapes the diversification of Carex (Cyperaceae)
Source: Ann Bot. 2025 Nov 20;137(3):777–88. doi: 10.1093/aob/mcaf290 (PMC12933657; doi:10.1093/aob/mcaf290)

Supplementary Figure S1. Set of 48 tested models representing hypotheses of different variables influencing diversification in *Carex*. *B1, B4, B7* and *B12* correspond to the studied bioclimatic variables (BIO1 -annual mean temperature-, BIO4 -temperature seasonality-, BIO7 -temperature annual range- and BIO12 -annual precipitation-, respectively). *LI* corresponds to lateral spike unit length, *CU* to culm length, *C2n* to chromosome number evolution, and *DI* to diversification rates. In the model code, the predictor influencing diversification is included after *d*.

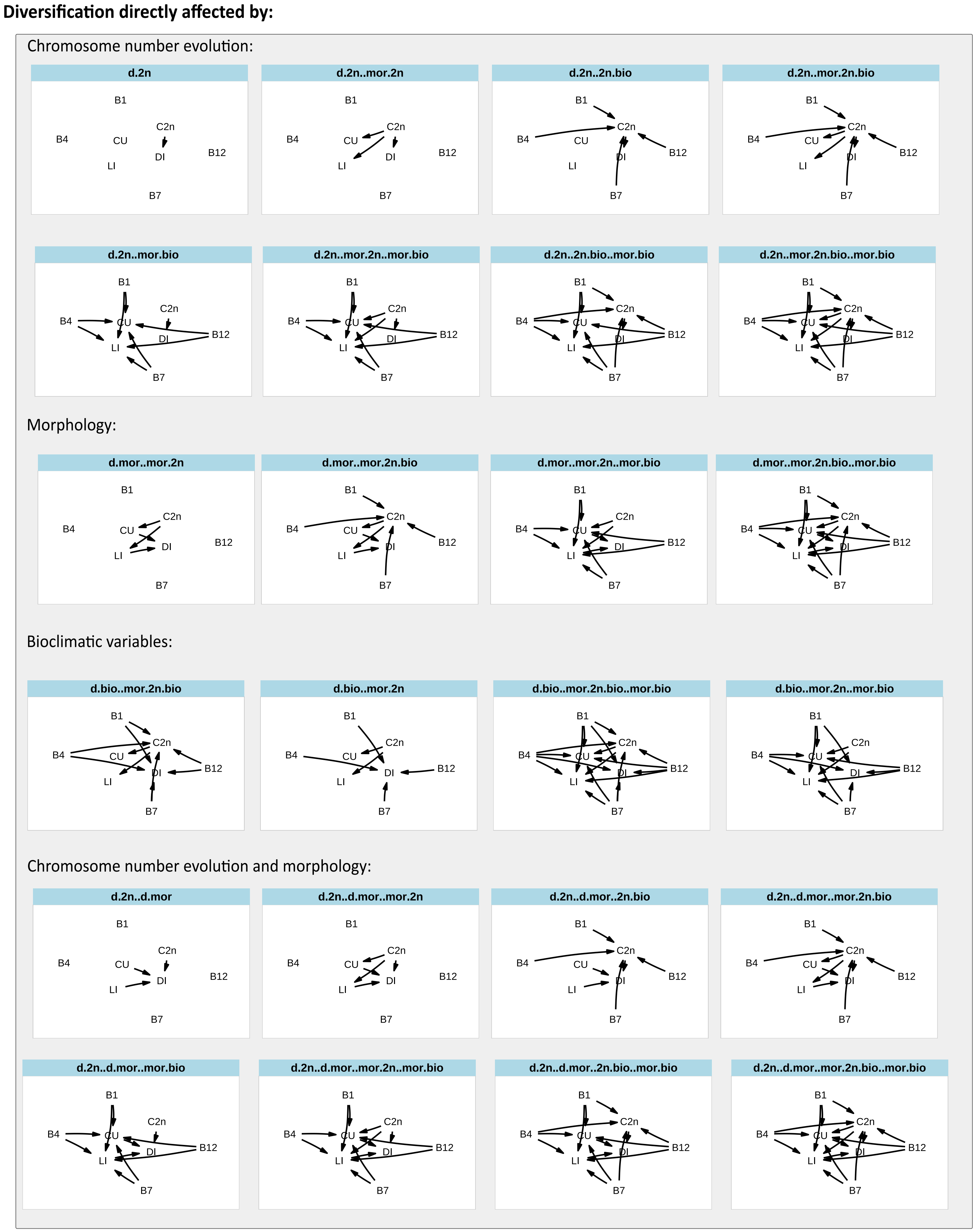

Chromosome number evolution and bioclimatic variables:

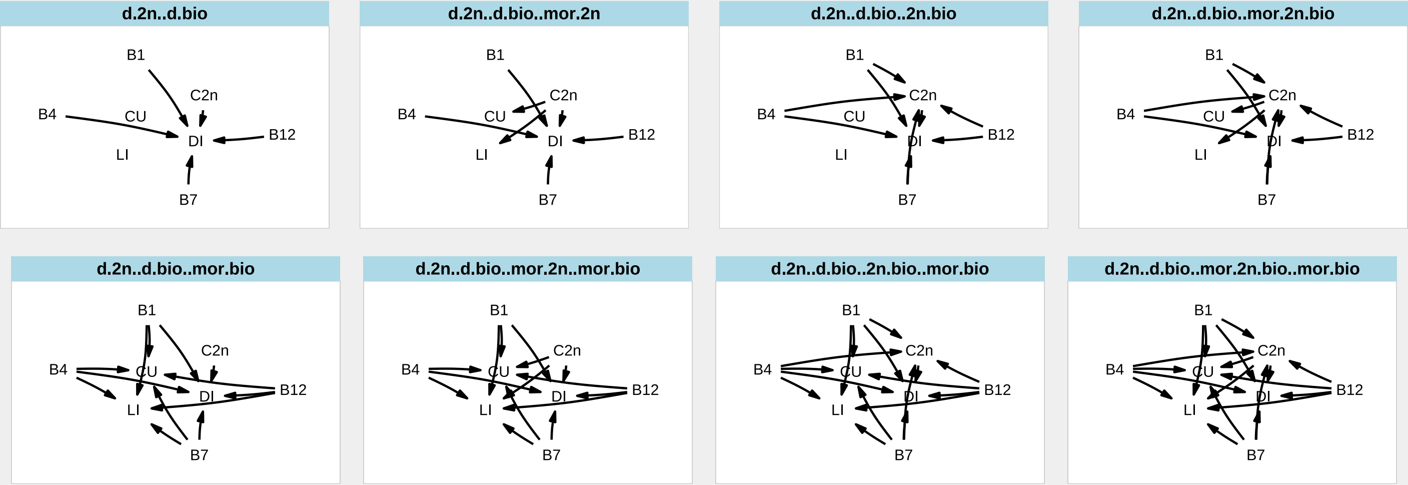

Bioclimatic variables and morphology:

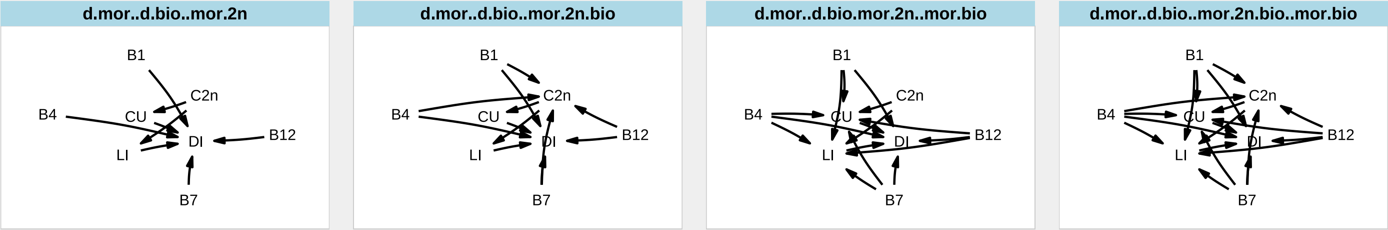

All:

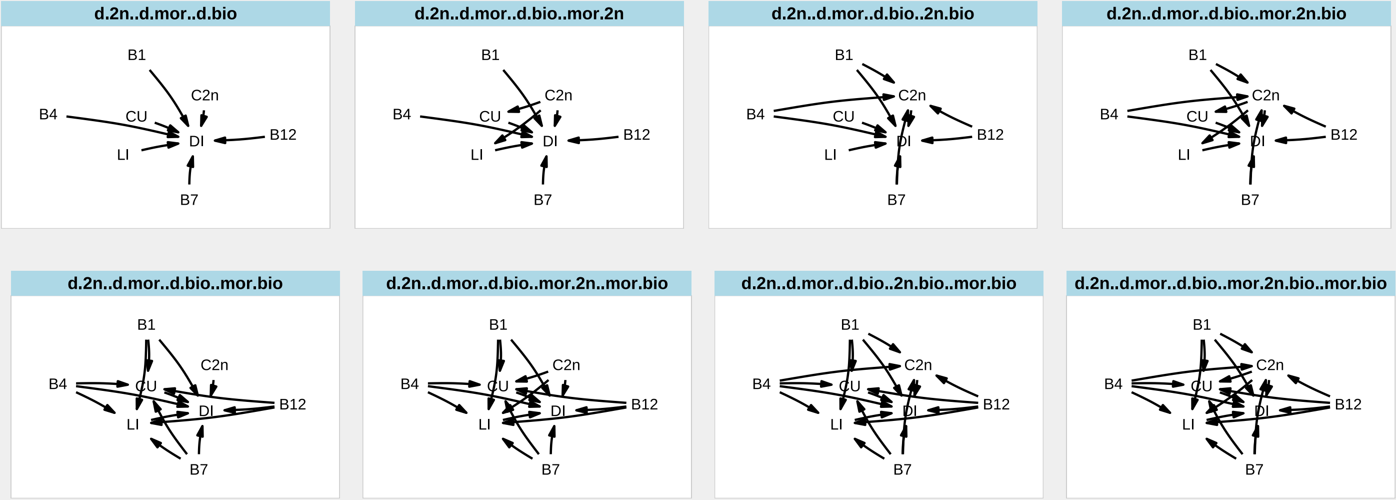

Diversification not affected by the studied variables

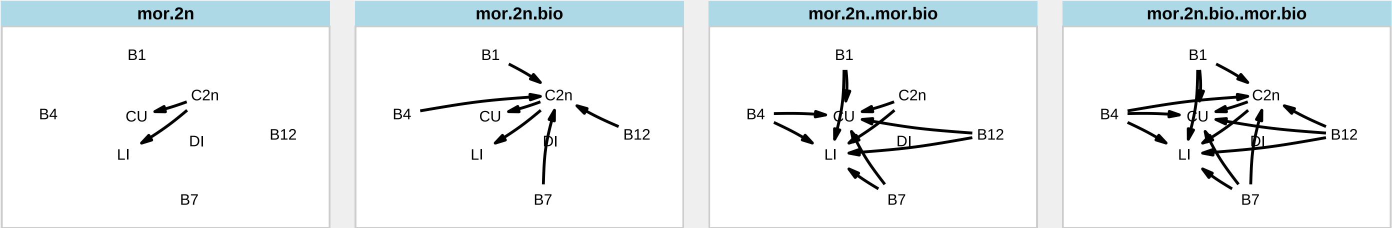

Supplementary Figure S2. Phylogeny showing shifts in evolutionary rates of bioclimatic variables from BAMM analysis: A) BIO1 (annual mean temperature), B) BIO4 (temperature seasonality), C) BIO7 (temperature annual range) and D) BIO12 (annual precipitation).

A

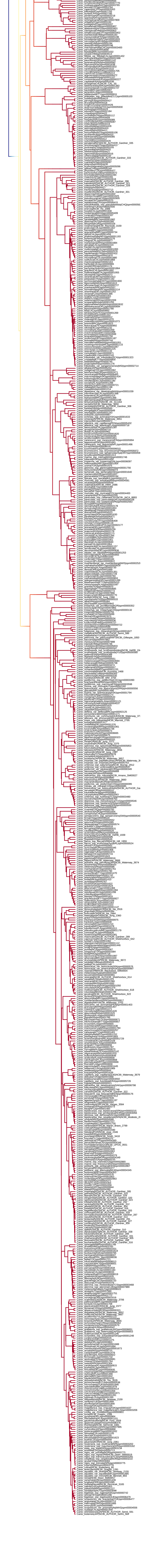

B

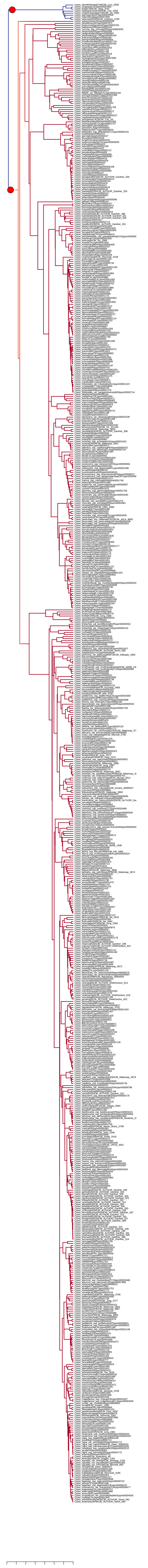

C

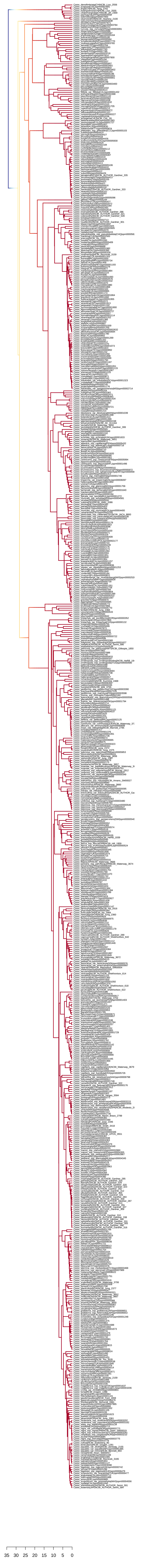

D

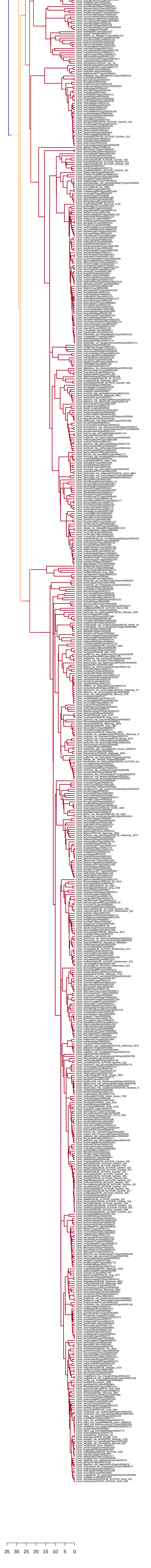

Supplementary Figure S3. Phylogeny showing shifts in the rate of culm evolution from BAMM analysis.

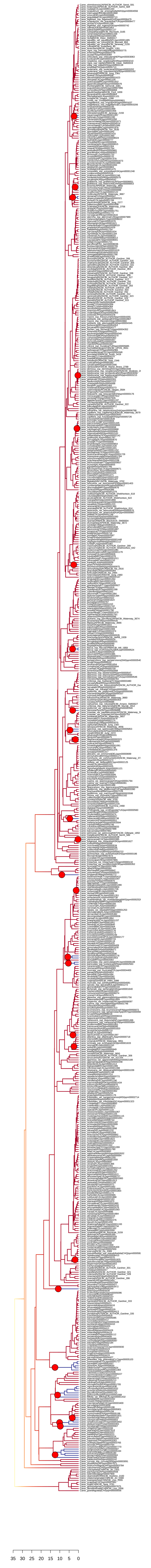

Supplementary Figure S4. Phylogeny showing shifts in the lateral unit length evolution from BAMM analysis.

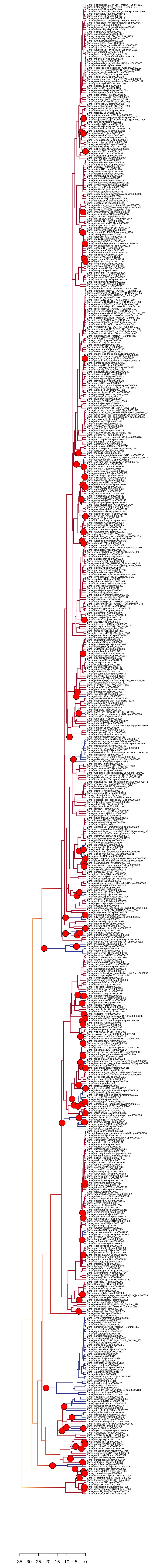

Supplementary Figure S5. (A) Models support using only evolutionary rates, assessed through phylogenetic path analyses. (B) Model support using only variables, assessed through phylogenetic path analyses, and (C) Model support using both variables and evolutionary rates, assessed through phylogenetic path analyses. Bars represent model weights, with color indicating whether models fall within 2 CICc units.

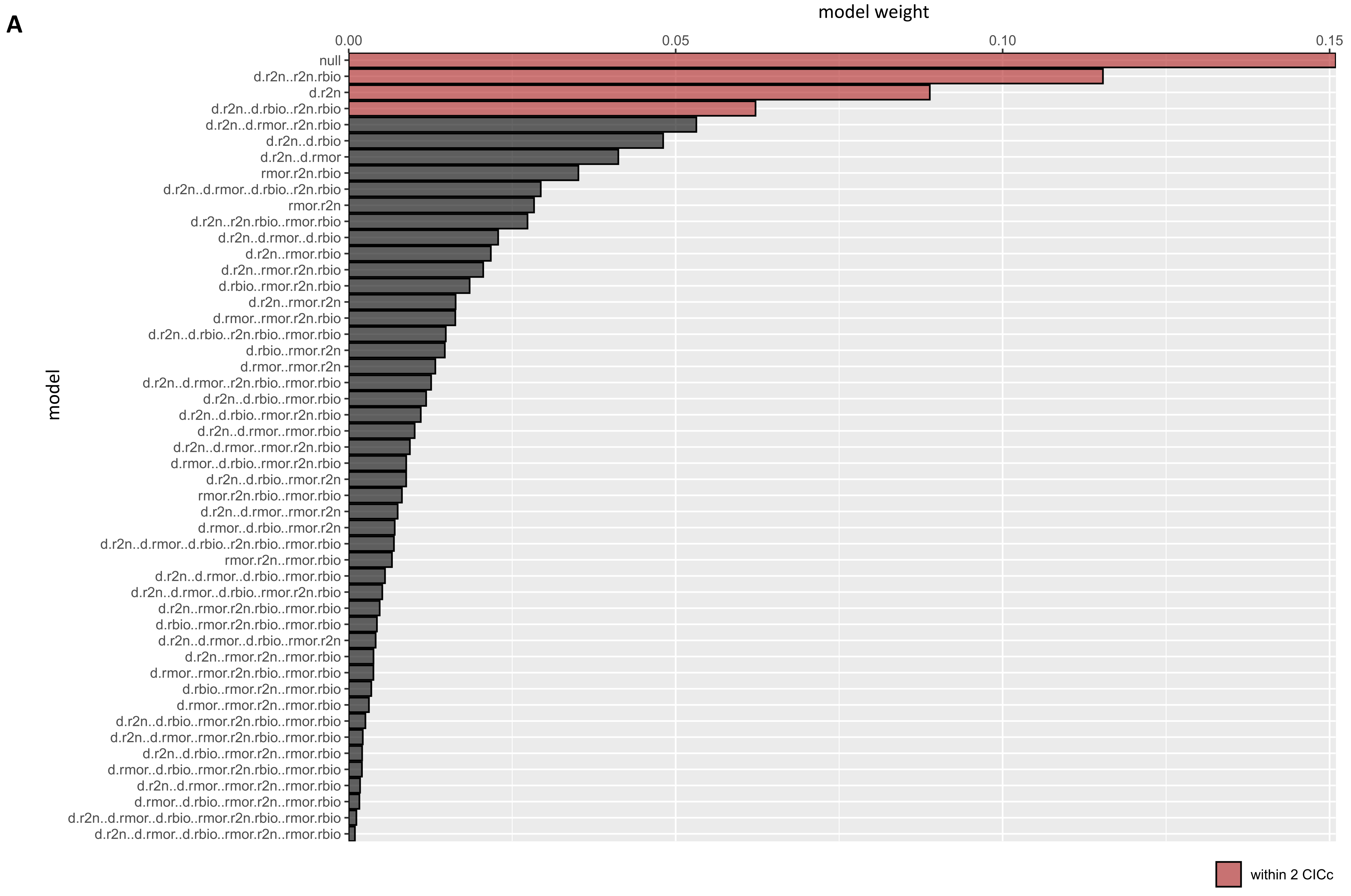

B

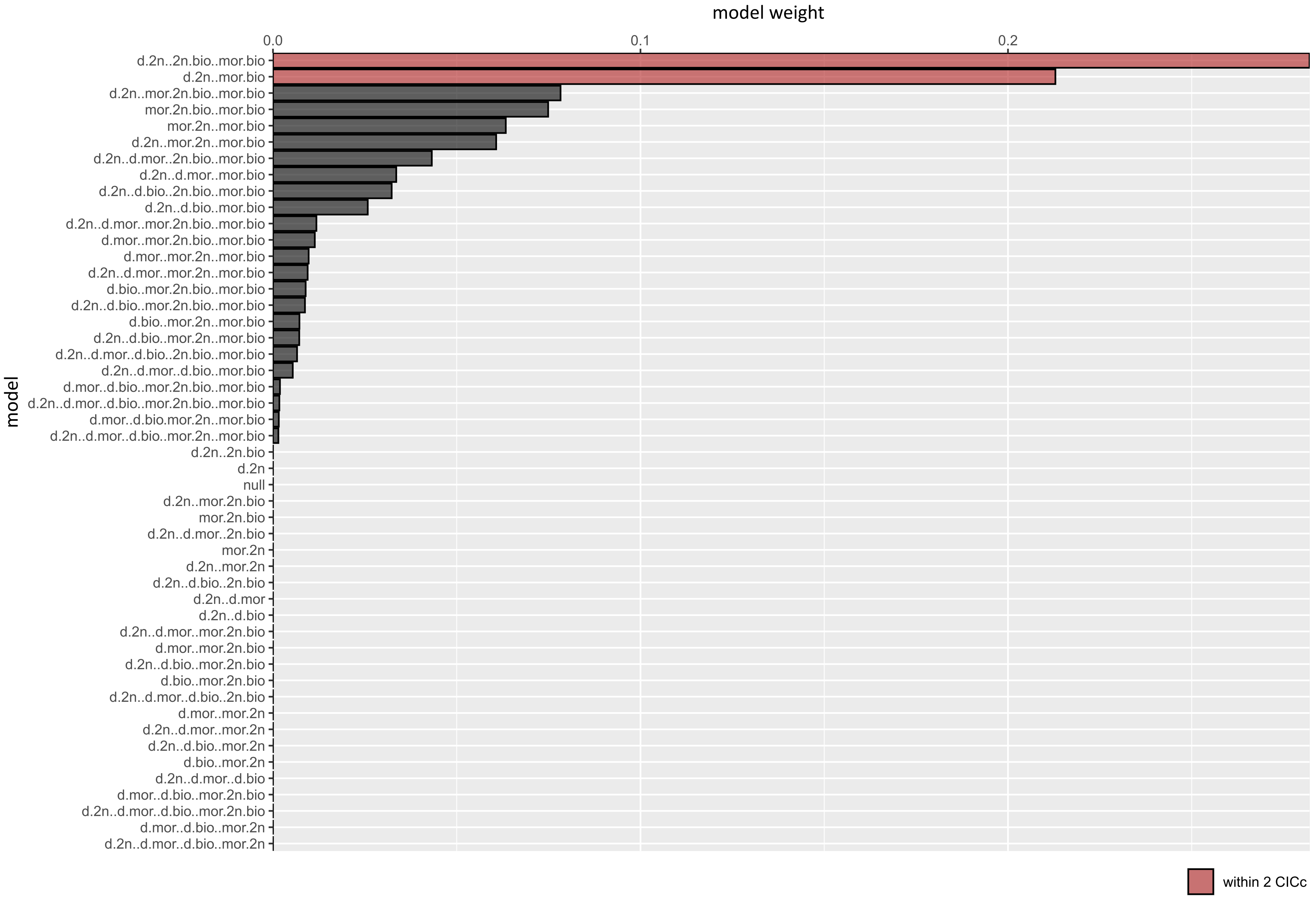
model weight
0.0
0.1
0.2
model
d.2n..2n.bio..mor.bio
d.2n..mor.bio
d.2n..mor.2n.bio..mor.bio
mor.2n.bio..mor.bio
mor.2n..mor.bio
d.2n..mor.2n..mor.bio
d.2n..d.mor..2n.bio..mor.bio
d.2n..d.mor..mor.bio
d.2n..d.bio..2n.bio..mor.bio
d.2n..d.bio..mor.bio
d.2n..d.mor..mor.2n.bio..mor.bio
d.mor..mor.2n.bio..mor.bio
d.mor..mor.2n..mor.bio
d.2n..d.mor..mor.2n..mor.bio
d.bio..mor.2n.bio..mor.bio
d.2n..d.bio..mor.2n.bio..mor.bio
d.bio..mor.2n..mor.bio
d.2n..d.bio..mor.2n..mor.bio
d.2n..d.mor..d.bio..2n.bio..mor.bio
d.2n..d.mor..d.bio..mor.bio
d.mor..d.bio..mor.2n.bio..mor.bio
d.2n..d.mor..d.bio..mor.2n.bio..mor.bio
d.mor..d.bio.mor.2n..mor.bio
d.2n..d.mor..d.bio..mor.2n..mor.bio
d.2n..2n.bio
d.2n
null
d.2n..mor.2n.bio
mor.2n.bio
d.2n..d.mor..2n.bio
mor.2n
d.2n..mor.2n
d.2n..d.bio..2n.bio
d.2n..d.mor
d.2n..d.bio
d.2n..d.mor..mor.2n.bio
d.mor..mor.2n.bio
d.2n..d.bio..mor.2n.bio
d.bio..mor.2n.bio
d.2n..d.mor..d.bio..2n.bio
d.mor..mor.2n
d.2n..d.mor..mor.2n
d.2n..d.bio..mor.2n
d.bio..mor.2n
d.2n..d.mor..d.bio
d.mor..d.bio..mor.2n.bio
d.2n..d.mor..d.bio..mor.2n.bio
d.mor..d.bio..mor.2n
d.2n..d.mor..d.bio..mor.2n
within 2 CICc

C

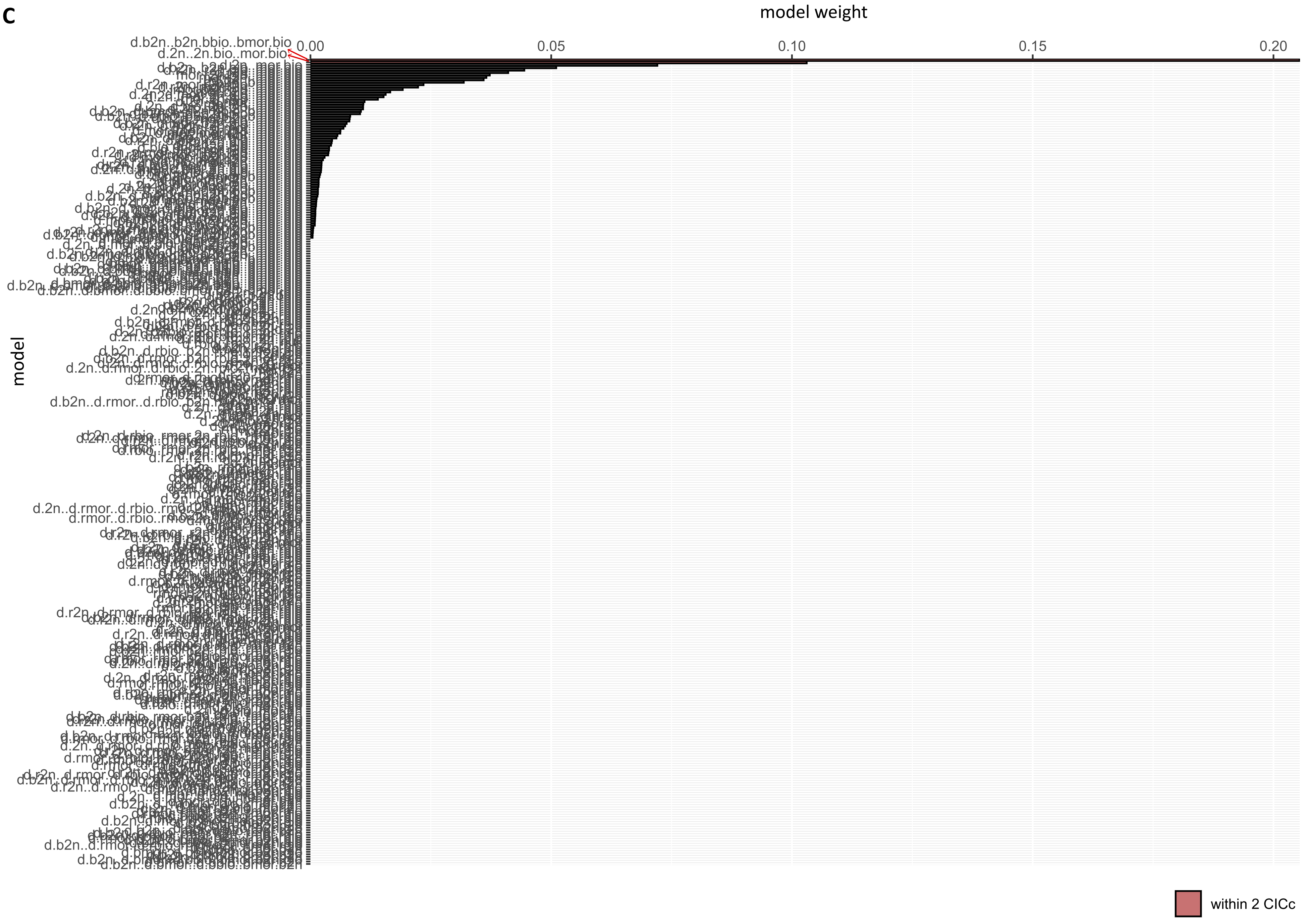
model weight
0.00
0.05
0.10
0.15
0.20
model
d.b2n..b2n.bbio..bmor.bio
d.2n..2n.bio..mor.bio
d.b2n..d.bmor..d.bbio..bmor.b2n
within 2 CICc

Supplementary Figure S6. Relationship between chromosome number evolution and speciation (λ), extinction (μ) and net diversification rates, inferred by the QuaSSE method.

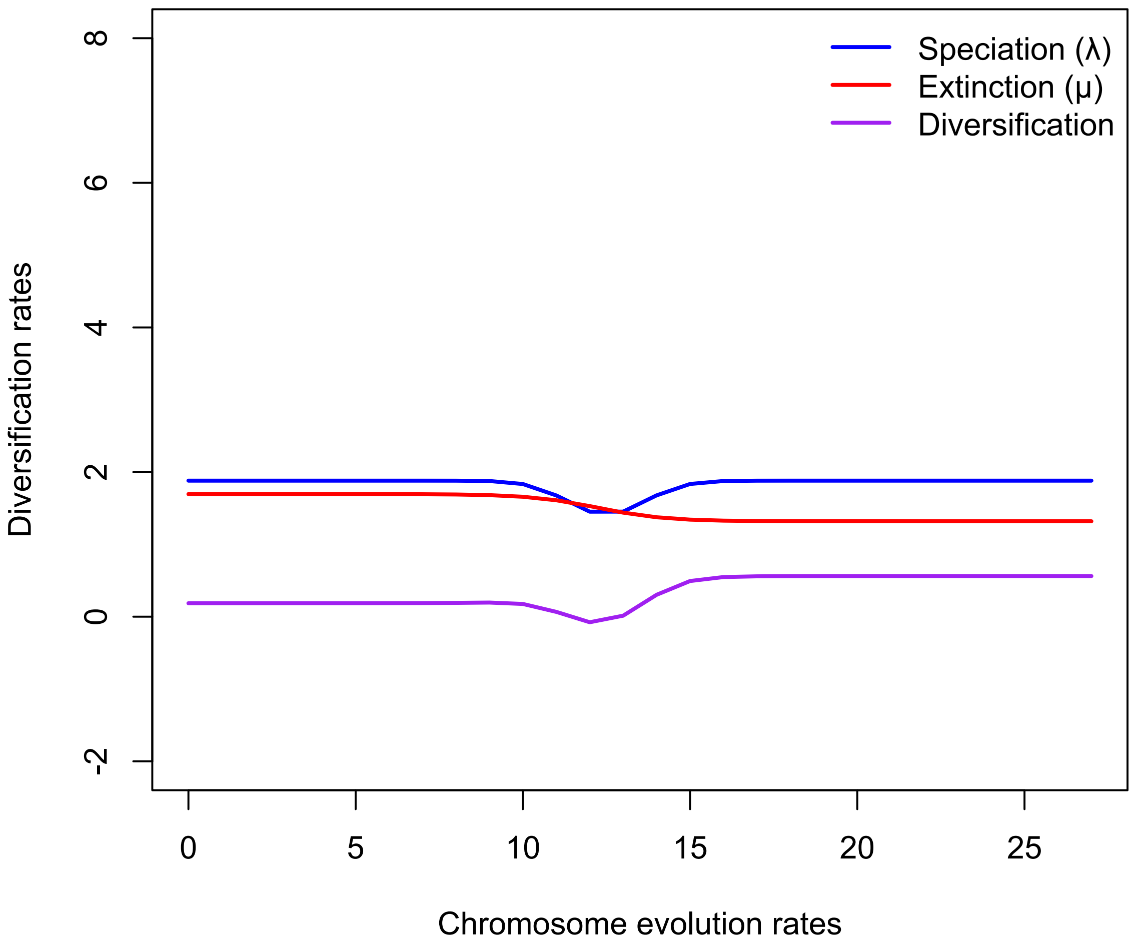

Supplement: mcaf290_Supplementary_Data [file mcaf290_supplementary_data.zip › Supplementary_figures_review2.pdf]
