# Supplementary material for: The complex interplay between chromosome, climatic niche and morphological traits shapes the diversification of Carex (Cyperaceae)
Source: Ann Bot. 2025 Nov 20;137(3):777–88. doi: 10.1093/aob/mcaf290 (PMC12933657; doi:10.1093/aob/mcaf290)
Supplement: mcaf290_Supplementary_Data [file mcaf290_supplementary_data.zip › Supplementary_tables.pdf]

Supplementary Table S1. Estimated coefficient for the full average best model among the 48 models with only rates of evolution, inferred through phylogenetic path analyses. *DI* corresponds to diversification rates, *R2n* represents the rate of chromosome evolution, *RCU* represents the evolutionary rate of the culm length, *RLI* corresponds to the evolutionary rate of the lateral spike unit length, and *RB4* represents the evolutionary rate of BIO4 (temperature seasonality). The highest coefficient is in bold font.

	DI	*R2n*	*RCU*	*RLI*	*RB4*
DI	0	0	0	0	0
R2n	-0.006 (-0.037-0.024)	0	0	0	0
RCU	0	0	0	0	0
RLI	0	0	0	0	0
RB4	0.004 (-0.041 – 0.048)	**0.046 (-0.120 - 0.212)**	0	0	0

Supplementary Table S2. Estimated coefficient for the full average best model among the 48 models with only variables of evolution, inferred through phylogenetic path analyses. *B1*, *B4*, *B7* and *B12* correspond to the studied bioclimatic variables (BIO1 -annual mean temperature-, BIO4 -temperature seasonality-, BIO7 -temperature annual range- and BIO12 -annual precipitation-, respectively). *LI* corresponds to lateral spike unit length, *CU* to culm length, *C2n* to chromosome number evolution, and *DI* to diversification rates. The highest coefficient is in bold font.

	B12	*B7*	*B4*	*B1*	*LI*	*CU*	*C2n*	*DI*
B12	0	0	0	0	0.010<br>(-0.076 - 0.095)	-0.093<br>(-0.184 - -0.002)	-0.020<br>(-0.080 - 0.039)	0
B7	0	0	0	0	0.007<br>(-0.280 - 0.294)	-0.025<br>(-0.327-0.277)	0.028<br>(-0.143 - 0.198)	0
B4	0	0	0	0	0.021<br>(-0.284 - 0.326)	0.180<br>(-0.138 -0.496)	-0.062<br>(-0.266 - 0.142)	0
B1	0	0	0	0	0.210<br>(0.102 - 0.317)	**0.484**<br>**(0.374 - 0.594)**	-0.005<br>(-0.067- 0.057)	0
LI	0	0	0	0	0	0	0	0
CU	0	0	0	0	0	0	0	0
C2n	0	0	0	0	0	0	0	-0.009<br>(-0.029 - 0.011)
DI	0	0	0	0	0	0	0	0

Supplementary Table S3. Estimated coefficient for the full average best model among the 332 models with both variables of evolution and their rates of evolution, inferred through phylogenetic path analyses. *B1, B4, B7* and *B12* correspond to the studied bioclimatic variables (BIO1 -annual mean temperature-, BIO4 -temperature seasonality-, BIO7 -temperature annual range-, and BIO12 -annual precipitation-), and *RB4* indicates the evolutionary rate of BIO4 -temperature seasonality-. *LI* and *RLI* correspond to lateral spike unit length and its evolution rate, respectively. *CU* and *RCU* indicate culm length and its evolution rate, respectively. Finally, *C2n* and *R2n* represent chromosome evolution and its rate, respectively; while *DI* indicates diversification rates. The highest coefficient is in bold font.

	B12	*B7*	*RB4*	*B4*	*B1*	*RLI*	*LI*	*RCU*	*CU*	*R2n*	*C2n*	*DI*
B12	0	0	0	0	0	0.00 (-0.056 - 0.056)	0.009 (-0.076-0.095)	0.005 (-0.044 - 0.054)	-0.094 (-0.185 - - 0.003)	-0.015 (-0.051 - 0.021)	-0.035 (-0.100 - 0.029)	0
B7	0	0	0	0	0	-0.072 (-0.288 - 0.144)	0.007 (-0.280-0.295)	0.018 (-0.145 - 0.181)	-0.025 (-0.327- 0.277)	-0.019 (-0.123 - 0.085)	0.044 (-0.173 - 0.262)	0
RB4	0	0	0	0	0	-0.028 (-0.332 - 0.276)	0.030 (-0.220 - 0.279)	-0.060 (-0.400 - 0.273)	0.045 (-0.135 - 0.224)	0.073 (-0.117- 0.264)	0.163 (-0.135 - 0.462)	0
B4	0	0	0	0	0	0.125 (-0.146 - 0.395)	0.021 (-0.285 - 0.327)	-0.031 (-0.214 - 0.152)	0.180 (-0.137 - 0.498)	0.009 (-0.101- 0.120)	-0.103 (-0.335 - 0.129)	0

	B12	*B7*	*RB4*	*B4*	*B1*	*RLI*	*LI*	*RCU*	*CU*	*R2n*	*C2n*	*DI*
B1	0	0	0	0	0	0.012 (-0.063 - 0.086)	0.212 (0.104 - 0.319)	-0.020 (-0.086 - 0.045)	**0.487 (0.377 - 0.597)**	0.013 (-0.029- 0.056)	-0.007 (-0.089 - 0.075)	0
RLI	0	0	0	0	0	0	0	0	0	0	0	0
LI	0	0	0	0	0	0	0	0	0	0	0	0
RCU	0	0	0	0	0	0	0	0	0	0	0	0
CU	0	0	0	0	0	0	0	0	0	0	0	0
R2n	0	0	0	0	0	0	0	0	0	0	0	0.011 (-0.048 - 0.069)
C2n	0	0	0	0	0	0	0	0	0	0	0	-0.014 (-0.049- 0.020)
DI	0	0	0	0	0	0	0	0	0	0	0	0

Supplementary Table S4. Model comparison results from QuaSSE analyses evaluating the relationship between trait evolution and diversification. The best-supported model based on AIC scores is in bold font.

	Df	*lnLik*	*AIC*	*ChiSq*	*Pr(>|Chi|)*
minimal	3	-2293.5	4593		
OU.constant.constant	4	-2292.6	4593.2	1.844	1.75E-01
BM.linear.constant	4	-2292.1	4592.2	2.762	9.65E-02
BM.constant.linear	4	-2292.8	4593.6	1.377	2.41E-01
OU.linear.constant	5	-2292.1	4594.2	2.762	2.51E-01
OU.constant.linear	5	-2291.9	4593.8	3.242	1.98E-01
BM.sigmoid.constant	6	-2289.1	4590.2	8.826	3.17E-02
OU.sigmoid.constant	7	-2288.9	4591.8	9.171	5.70E-02
BM.constant.sigmoid	6	-2290.7	4593.4	5.640	1.31E-01
OU.constant.sigmoid	7	-2289.2	4592.4	8.596	7.20E-02
BM.hump.constant	6	-2286.8	4585.6	13.454	3.75E-03
OU.hump.constant	7	-2286.8	4587.6	13.454	9.26E-03
BM.constant.hump	6	-2288.7	4589.4	9.651	2.18E-02
OU.constant.hump	7	-2288.7	4591.4	9.651	4.67E-02
BM.hump.linear	7	-2285.3	4584.7	16.331	2.61E-03
BM.hump.sigmoid	9	-2282.7	4583.3	21.708	1.37E-03
BM.linear.linear	5	-2293.3	4596.7	0.323	8.51E-01
BM.linear.sigmoid	7	-2289.6	4593.2	7.788	9.97E-02
BM.sigmoid.linear	7	-2289.1	4592.2	8.855	6.48E-02
BM.sigmoid.sigmoid	9	-2289.2	4596.4	8.641	1.95E-01
OU.hump.linear	8	-2283.2	4582.5	20.525	9.96E-04
OU.hump.sigmoid	**10**	**-2264**	**4548.1**	**58.919**	**2.48E-10**
OU.linear.linear	6	-2292.7	4597.4	1.567	6.67E-01
OU.linear.sigmoid	8	-2289.3	4594.5	8.488	1.31E-01
OU.sigmoid.linear	8	-2289.1	4594.2	8.855	1.15E-01

	Df	*lnLik*	*AIC*	*ChiSq*	*Pr(>|Chi|)*
OU.sigmoid.sigmoid	10	-2288	4595.9	11.072	1.36E-01
